# A Randomized Controlled Trial Assessing Full-Mouth Versus Quadrant-Based Scaling and Root Planing for Non-surgical Periodontal Therapy

**DOI:** 10.7759/cureus.82336

**Published:** 2025-04-15

**Authors:** Divya Gaddam, Sreenivas Nagarakanti, P. Sravya Sri, Bhagyasri Chiruvella, Rekha R, Tejasri Gudur

**Affiliations:** 1 Department of Periodontology, Narayana Dental College and Hospital, Nellore, IND

**Keywords:** full mouth disinfection, non surgical periodontal treatment, periodontitis, root planning, scaling

## Abstract

Aims and objectives

Mechanical debridement using subgingival Scaling and Root Planning (SRP) can unequivocally be considered a well-established and effective method for non-surgical treatment of periodontitis. The objective of the present study was to assess the clinical efficiency of Full Mouth Scaling (FMS) and Full Mouth Disinfection (FMD) compared to Quadrant-wise Scaling and Root Planing (Q-SRP) in patients with periodontitis.

Materials and methods

A randomized clinical trial was conducted among 180 patients having periodontitis. The patients were randomly divided into three treatment groups. Group I received Full Mouth Scaling (FMS), Group II received Quadrant-wise Scaling and Root Planning (Q-SRP), Group III received Full Mouth Disinfection (FMD). Assessment of clinical parameters was done at baseline and 3 months.

Results

Intragroup analysis revealed a statistically significant difference among all three groups from baseline to three months (p ≤0.05). Intergroup comparisons revealed no significant differences in terms of Probing Pocket Depth (PPD) and Clinical Attachment Level (CAL) among the three groups. During the study period, Plaque Index (PI) and modified Sulcus Bleeding Index (mSBI) changes were reduced significantly in Q-SRP compared to the other two groups. Operator fatigue was comparatively less in Q-SRP compared to FMS and FMD.

Conclusions

The presented results depict the short-term effects. All three treatment modalities showed improvements in clinical parameters after three months. Each of the three treatment strategies shows effectiveness and can be recommended for managing severe chronic periodontitis.

## Introduction

Periodontitis is a widespread inflammatory condition affecting tissues that support the teeth, potentially resulting in tooth loss if not addressed [1]. It often begins with gingivitis, a milder gingival inflammation, but can advance to a more severe form, causing damage to the periodontium. Without treatment, this disease can create deep pockets between the teeth and gingiva, leading to further bone loss. Various reasons such as poor oral hygiene, smoking, diabetes, genetics, and certain medications increase the risk of periodontitis. early intervention through professional dental treatment plays a vital role in preventing long-term damage.

The standard initial intervention in treating chronic periodontitis is non-surgical periodontal therapy (NSPT), which mainly includes Scaling and Root Planing (SRP) [2,3]. SRP is aimed to remove the plaque and calculus not only supra-gingivally but also sub-gingivally. This initial treatment restores gingival health by reducing inflammation. Two alternative strategies have been proposed to improve the effectiveness and outcomes of NSPT: Full Mouth Disinfection (FMD) and Full Mouth Scaling (FMS).

FMD includes SRP of all teeth within a duration of 24 hours, followed by using antimicrobial agents like 0.2% chlorhexidine for a period of two weeks postoperative, with the aim to swiftly lower the bacterial load [4,5]. FMS is similar but does not include the use of antimicrobials. In contrast, the conventional approach, Quadrant-wise Scaling and Root Planing (Q-SRP), involves SRP in each quadrant separately over multiple appointments [6,7].

A group of researchers in their series of studies initially found that full-mouth SRP was more effective than conventional quadrant SRP in managing periodontal disease. Their findings showed that the full-mouth approach led to superior clinical and microbiological outcomes, even without the use of chlorhexidine as an adjunct treatment [8].

However, more recent studies from different research centers have failed to show any clear benefit of performing FMS within a duration of 24 hours (hrs), as compared to the Q-SRP approach [9-18]. In supporting this research, this study is aimed evaluate the clinical outcomes of FMD, FMS, and Q-SRP in the treatment of periodontitis.

## Materials and methods

Ethical approval

This study was accepted by the Institutional Ethical Committee (reference number IEC/NDCH/2022/Mar/P-05) of Narayana Dental College and Hospital, Nellore, Andhra Pradesh, India, and the study was registered in the Clinical Trials Registry of India (Reference id: CTRI/2022/04/042316).

Sample size estimations

The sample size was determined using G*Power analysis (Heinrich Heine University, Dusseldorf, Germany) with an effective size of 0.25, α value at 0.05, and the power of this study was kept at 80%. The total sample size achieved was 159 (53 per group). Considering the dropouts or attrition rate of 10% (N=6), 180 participants were included (60 per group).

Participants and study design

This study was conducted as a parallel-group randomized, double-blinded (data collector and data analyst) clinical trial with three groups, each having an equal allocation ratio of 1:1:1. The study subjects were recruited from patients who visited the Department of Periodontology between July 2022 to January 2023.

**Figure 1:**
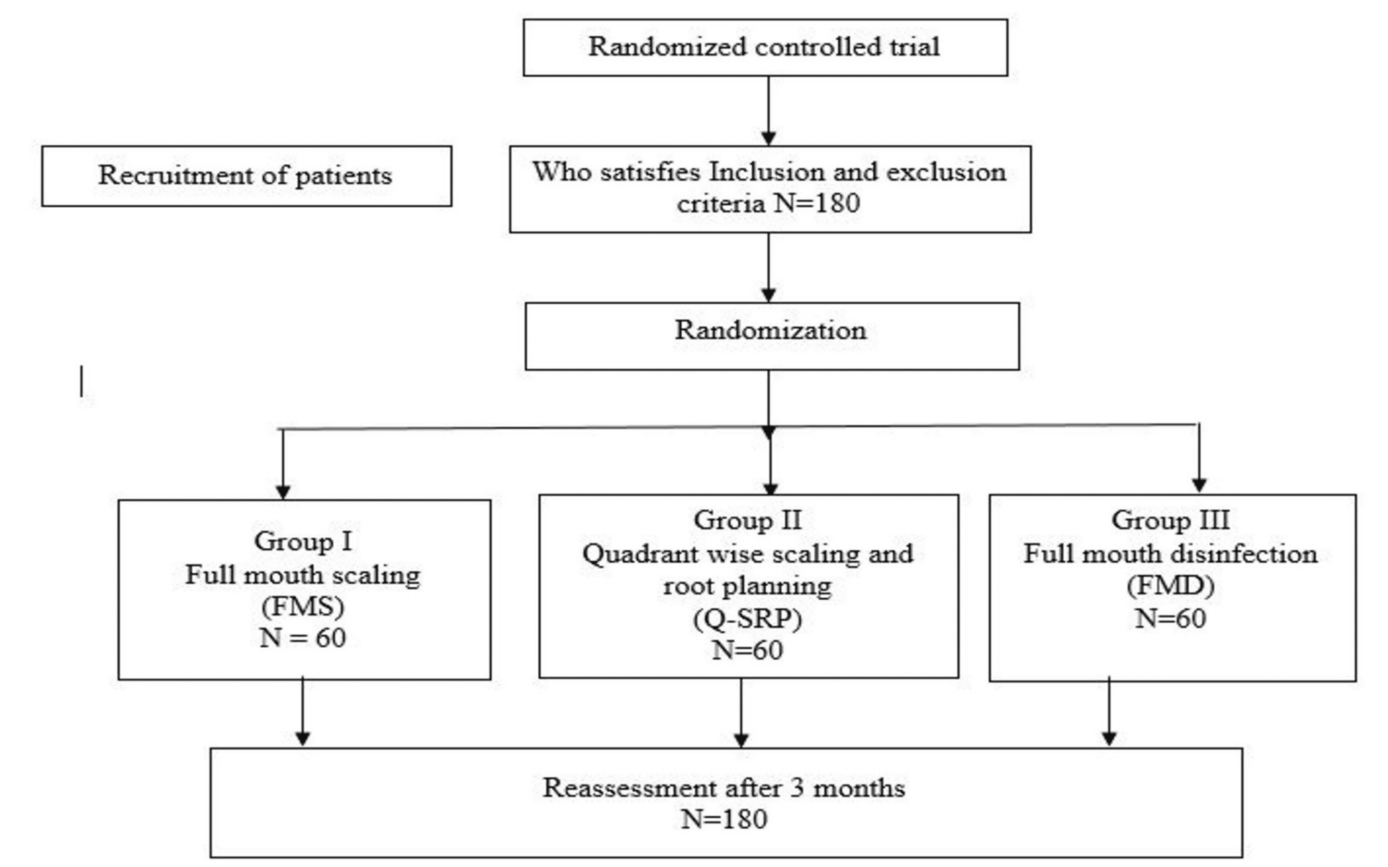
CONSORT flow chart of the study CONSORT: Consolidated Standards of Reporting Trials; N: number of participants

All participants were informed about the study's purpose, nature, and design, and written consent was obtained prior to their participation. Computer-generated randomization was then performed, and participants were assigned to one among the three treatment groups, as outlined in the study flowchart (Figure 1): FMS: Full-mouth SRP within a time of 24 hrs, without the usage of antiseptics; Q-SRP: Quadrant-wise SRP done in clockwise in four sessions, with an interval of 1 week for each quadrant, without the use of antiseptics; and FMD: Full-mouth SRP within 24 hrs, done with disinfection using the antiseptic application according to the protocol given by Quirynen et al. [19] - brushing the dorsum of the tongue with 1% chlorhexidine gel for 1 min, rinsing mouth twice with 0.2% chlorhexidine solution for 1 min, twice application of 0.2% chlorhexidine spray to the tonsils, subgingival irrigation of all pockets with 1% chlorhexidine gel (three times within 5-10 min), which was repeated after 1 week; patients were instructed to rinse mouth twice a day for 1 min with 0.2% chlorhexidine solution and to spray the tonsils twice a day with 0.2% chlorhexidine spray over a period of 2 months.

Inclusion and exclusion criteria

Systemically healthy individuals with stage II/III periodontitis, the presence of a minimum of 18 teeth, with a probing depth of ≥ 5mm in at least six sites with radiographic bone loss were included in the study. Patients who had undergone SRP in the preceding 6 months or using antimicrobial rinsing solutions or consuming systemic antibiotics within the last 4 months, who had systemic diseases that are known to have interactions with periodontal diseases or require antibiotic prophylaxis, intake of drugs with possible impact on clinical symptoms of periodontal diseases, pregnancy and patients who are using tobacco in any form were excluded.

In all treatment groups, subgingival instrumentation was performed after local anesthesia and included the use of ultrasonic scalers (Piezon Master®, EMS, Nyon, Switzerland), followed by instrumentation with Gracey curettes (Hu-Friedy, Frankfurt, Germany). All treatments were performed by an experienced therapist who was calibrated 3 months in advance to the start of the study. Postoperative instructions and appropriate brushing techniques were advised to all the participants. Patients were asked whether they experienced any kind of health impairment (e.g., systemic reactions, allergies, or increased temperature) in the post-treatment period.

Each of the sites was assessed for the following clinical parameters at baseline and 3 months: Plaque Index (PI), modified Sulcus Bleeding Index (mSBI), Probing Pocket Depth (PPD), and Clinical Attachment Level (CAL) as the primary outcomes. Visual Analogue Scale (VAS) to assess pain and discomfort, and Operator Fatigue Scale (OFS) were the secondary outcomes.

Statistical analysis

Descriptive statistics were employed to gain an initial understanding of the dataset. For continuous variables, mean and standard deviation (SD) were assessed, while categorical or dichotomous variables were summarized using percentages. In hypothesis testing, one-way ANOVA and post hoc Bonferroni correction tests were used to compare means between the three groups. Specifically, one-way ANOVA tests were employed to assess differences in continuous variables between groups. A significance level of 0.05 was set for all hypothesis tests, with results measured to be statistically significant if the p-value was less than 0.05. These analyses were carried out using the R-based Jamovi software to ensure a thorough and systematic exploration of the study's findings [20,21,22].

## Results

The study consisted of three groups (FMS, Q-SRP, and FMD), each with 60 participants. None of the three groups' participants experienced any negative side effects from the treatments they received. The mean ages of FMS, Q-SRP, and FMD were 46.4, 47.2, and 48.4 years, respectively. The Q-SRP group had the widest age range, while the FMD group had the highest average age. The characteristics of the patient and the demographic data are given in Table 1.

**Table 1:** Demographic data N - Total number of participants in each group; FMS - Full Mouth Scaling; Q-SRP - Quadrant-wise Scaling and Root Planing; FMD - Full Mouth Disinfection

Group	Number (N)	Mean±SD Age in Years
FMS	60	46.4±7.61
Q-SRP	60	47.2±9.21
FMD	60	48.4±9.08

Table 2 presents intergroup comparisons of baseline variables across the FMS, Q-SRP, and FMD groups. No statistically significant differences were found for the PI between groups (p > 0.05). Significant differences were observed in mSBI between FMS and both Q-SRP and FMD, while PPD and CAL comparisons mostly showed non-significant differences, except for CAL between FMS and FMD, which was significant. These findings highlight key baseline differences, emphasizing the need to consider both statistical and clinical relevance.

**Table 2:** Intergroup comparison of periodontal parameters at baseline *P-value <0.05 considered statistically significant PI: Plaque Index; mSBI: modified Sulcular Bleeding Index; PPD: Probing Pocket Depth; CAL: Clinical Attachment Level; FMS: Full Mouth Scaling; FMD: Full Mouth Disinfection; Q-SRP: Quadrant-wise Scaling and Root Planing

Parameters	Inter-group comparison		Mean Difference	F statistics	P value	One-Way ANOVA
PI	FMS	Q-SRP	-0.16 (-0.36, 0.03)	6.711	0.13	P < 0.05*
		FMD	-0.14 (-0.33, 0.05)		0.25	
	Q-SRP	FMS	0.16 (-0.03, 0.36)		0.13	
		FMD	0.02(-0.17, 0.22)		1.00	
	FMD	FMS	0.14 (-0.05, 0.33)		0.25	
		Q-SRP	-0.02 (-0.22, 0.17)		1.00	
mSBI	FMS	Q-SRP	-0.13 (-0.29, 0.01)	9.441	0.10	
		FMD	-0.17(-0.33,-0.01)		0.02*	
	Q-SRP	FMS	0.13 (-0.01, 0.29)		0.10	
		FMD	-0.03(-0.19, 0.12)		1.00	
	FMD	FMS	0.17 (0.01, 0.33)		0.02*	
		Q-SRP	0.03 (-0.12, 0.19)		1.00	
PPD	FMS	Q-SRP	0.01 (-0.49, 0.52)	.036	1.00	
		FMD	-0.13 (-0.64, 0.37)		1.00	
	Q-SRP	FMS	-0.01 (-0.52, 0.49)		1.00	
		FMD	-0.15 (-0.66, 0.35)		1.00	
	FMD	FMS	0.13 (-0.37, 0.64)		1.00	
		Q-SRP	0.15 (-0.35, 0.66)		1.00	
CAL	FMS	Q-SRP	0.09 (-0.30, 0.49)	.803	1.00	
		FMD	-0.17 (-0.57, 0.22)		0.9	
	Q-SRP	FMS	-0.09 (-0.49, 0.30)		1.00	
		FMD	-0.26 (-0.66, 0.13)		0.33	
	FMD	FMS	0.17 (-0.22, 0.57)		0.90	
		Q-SRP	0.26 (-0.13, 0.66)		0.33	

**Figure 2:**
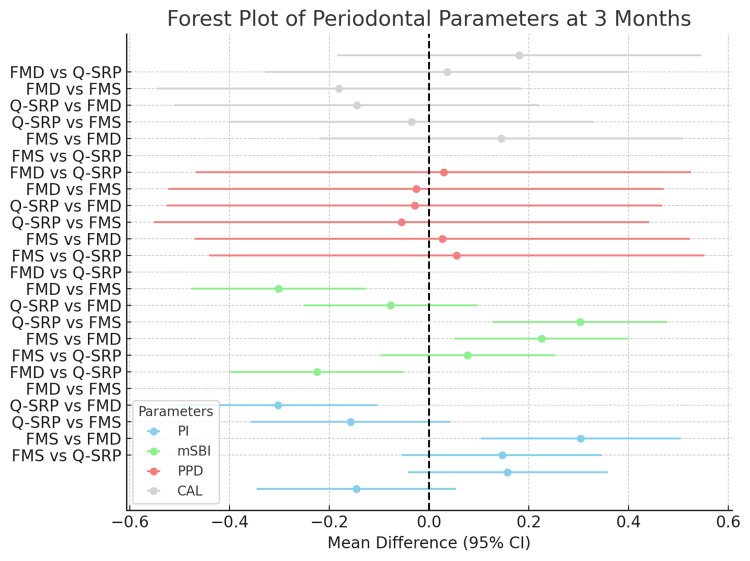
Forest plot of periodontal parameters at 3 months FMS: Full Mouth Scaling; FMD: Full Mouth Disinfection; Q-SRP: Quadrant-wise Scaling and Root Planing

Intergroup comparisons at 3 months for the FMS, Q-SRP, and FMD groups are shown in Figure 2. No statistically significant differences in PI were found between FMS and Q-SRP or FMS and FMD, but a significant variance was observed between Q-SRP and FMD, indicating variations in periodontal health. Significant differences in the mSBI were noted between FMS and Q-SRP, and between Q-SRP and FMD. PPD showed no significant differences across groups, while CAL differences were significant only between FMD and Q-SRP. These results highlight the need for tailored interventions based on group-specific periodontal differences.

Table 3 reports intergroup comparisons for the VAS and OFS among the FMS, Q-SRP, and FMD groups. Significant differences in the VAS scale were observed between FMS and Q-SRP; Q-SRP and FMS and FMD; reflecting variations in subjective experiences. However, no significant variations were found among FMS and FMD. For the OFS, all pairwise comparisons showed significant differences, indicating varying levels of operator fatigue among the groups. FMS participants reported higher fatigue than those in Q-SRP but lower than those in FMD. These findings highlight distinct subjective experiences and fatigue levels across the groups.

**Table 3:** Intergroup comparison of secondary outcomes VAS and OFS *P-value <0.05 considered statistically significant VAS: Visual Analogue Scale; OFS: Operator Fatigue Scale; FMS: Full Mouth Scaling; FMD: Full Mouth Disinfection; Q-SRP: Quadrant-wise Scaling and Root Planing

Scales	Inter-group comparison		Mean Difference	F statistics	P-value	One-Way ANOVA
VAS	FMS	Q-SRP	-1.35	60.253	0.001^*^	P < 0.05*
		FMD	-0.15		0.800	
	Q-SRP	FMS	1.35		0.001^*^	
		FMD	1.2		0.001^*^	
	FMD	FMS	0.15		0.800	
		Q-SRP	-1.2		0.001^*^	
OFS	FMS	Q-SRP	1.45	93.141	0.001^*^	
		FMD	-.76		0.001^*^	
	Q-SRP	FMS	-1.45		0.001^*^	
		FMD	-2.21		0.001^*^	
	FMD	FMS	.76		0.001^*^	
		Q-SRP	2.21		0.001^*^	

## Discussion

Dental plaque is a main etiological factor in the causation of periodontal disease, a multifaceted infection driven by specific anaerobic Gram-negative bacteria that result in the destruction of the tissues supporting the teeth [23]. This disease is influenced by two main elements: the production of harmful substances by these microorganisms and the inflammatory response from the host, which releases cytokines, interleukins, and metalloproteinases that further contribute to tissue destruction. Traditional treatment approaches focus on the mechanical removal of bacterial deposits, both above and below the gingiva, and educating patients on oral hygiene to manage inflammation and prevent tissue destruction.

In the current periodontal therapy, SRP is the standard procedure used and is typically carried out over several weeks. Studies comparing full-mouth treatments, such as FMD and FMS, to conventional SRP have yielded mixed results. While some studies have exhibited benefits in terms of reducing pocket depth and improving clinical attachment, these differences are often considered too small to be clinically significant. Systematic reviews, including those by Lang et al. and Teughels et al., suggest that although full-mouth treatments might offer certain improvements, the evidence remains inconsistent [6,24].

Additional analyses by Tunkel et al., van der Weijden and Timmerman, and Hallmon and Rees suggest that both hand- and power-driven instruments are equally effective for periodontal debridement. Tunkel et al. observed that ultrasonic devices tend to reduce treatment time, while Hallmon and Rees noted insufficient evidence to support this claim. Despite differences in conclusions about treatment duration, the consensus is that both types of instruments are effective in achieving comparable clinical outcomes [25-27].

Recent studies have assessed the effectiveness of FMS, FMD, and Q-SRP for treating periodontitis. These findings indicated that all three approaches led to improvements in clinical measurements from baseline to three months [28]. One systematic review noted only minor differences between FMD and Q-SRP in the treatment of chronic periodontitis among adults [13]. Another systematic review concluded that neither FMD nor FMS offered clinical benefits over the conventional strategy [6].

When comparing FMS and Q-SRP, similar impacts were observed on plaque levels, probing depth, and clinical attachment levels in this study. However, Q-SRP demonstrated a significant decrease in bleeding on probing. In comparisons between Q-SRP and FMD, significant differences were noted in plaque index and bleeding on probing, with Q-SRP showing better outcomes. No significant differences were found in probing depth and clinical attachment levels between these methods.

Additionally, the study assessed patient discomfort using the VAS and operator fatigue using the OFS finding that FMS resulted in less perceived discomfort compared to both Q-SRP and FMD. These findings suggest that FMS might be more favorable in terms of patient comfort and operator fatigue, although further research is needed to thoroughly explore these factors.

Overall, while the efficacy of full-mouth and quadrant-wise treatments appears comparable, considerations of patient comfort and operator fatigue could influence treatment choices. Future studies should incorporate these aspects to provide a more comprehensive understanding of periodontal treatment outcomes.

The study has limitations that could impact its findings. Firstly, a larger sample size would have enhanced the robustness of the results. Additionally, extending the follow-up period beyond three months and incorporating multiple visits might have provided a more comprehensive view of long-term treatment effects. The study also did not account for other clinical parameters, such as tooth mobility and furcation involvement, which could influence overall plaque control. Moreover, including microbiological and biological assessments could have offered more detailed insights into the treatment outcomes. These factors highlight the need for further research with broader sample sizes, extended follow-up periods, and a more holistic approach to clinical and biological parameters.

## Conclusions

The study underscores the value of personalized periodontal treatment, noting that customized care can improve patient outcomes. Within the limitations of this study, such as the sample size and follow-up period, all three treatment methods - FMS, Q-SRP, and FMD - showed notable improvements. These results indicate that each approach is effective for addressing severe periodontitis, giving clinicians a variety of strategies that can be adjusted to suit the unique needs and circumstances of individual patients.
